# Can cashew nut allergy resolve spontaneously?

**DOI:** 10.1002/clt2.12385

**Published:** 2024-08-13

**Authors:** Tamazoust Guiddir, Audrey Siberil, Françoise Lepape, Marion Hacker, Ariane Nemni

**Affiliations:** ^1^ Pulmonology and Allergology Pediatric Unit AP‐HP Bicêtre Hospital Le Kremlin Bicêtre France; ^2^ Allergology Practice Maisons‐Laffitte France; ^3^ Children and Adult Allergology Department Robert Ballanger Hospital Aulnay‐sous‐Bois France

**Keywords:** cashew nut allergy, children, natural history, recombinant Ana o 3, recovery

To the Editor

Cashew nut allergy (CNA) is increasing worldwide and is responsible for severe anaphylaxis, particularly in young children.^1^ Symptoms range from mild reactions to severe anaphylaxis. The three main allergens are storage proteins: Ana o 1, Ana o 2 (cupin superfamily) and Ana o 3 (prolamin superfamily).^2^ Lifetime avoidance of cashew nut is currently recommended for those with CNA. However, little is known about the natural history of CNA.

We report a cohort of five children with severe anaphylaxis to cashew nut who recovered and were able to eat cashew nut after a successful oral food challenge (Table 1). They all presented severe anaphylaxis according to the ordinal food allergy severity score (oFAAS‐5)^3^ (grade 3 to grade 5) at diagnosis at a mean age of 3 years [1.5–4]. Two patients had no atopy, one had a personal and familial atopic history and two others had only personal atopy. They all had no allergies or sensitizations to peanuts or tree nuts. Three patients consumed native cashew (between one and three cashew units) during the first reaction and two patients consumed cashew in cooked meals (unknown quantity). Allergology explorations were performed a mean 1.1 years [0.15–5] after the first reaction. All patients were sensitized to pistachio, but only two had a confirmed food allergy to pistachio. Skin prick tests (SPTs) were performed with commercial extract (ALK‐Abello) and were deemed positive when wheal size was ≥ 3 mm. Cashew SPTs were positive for four children (mean 6 mm, range [3–20]). Cashew‐specific IgEs (ImmunoCap® by Phadia 1000 System, Thermo Fisher Scientific) were positive for all patients, with a mean of 1 KU/L [0.36–2.49]. The recombinant Ana o 3 was not tested for at diagnosis for three patients and was positive for two of them (0.63 and 1.97 KU/L). After the reaction, they all observed strict avoidance of cashew and pistachio in their diet, without any recurrence. During a mean follow‐up of 2.4 years (range [1–4]), the SPT and the cashew‐specific IgEs became negative (Figure 1) and all patients tested negative for recombinant Ana o 3. An oral food challenge in four patients was successful at a cashew nut cumulated dose of 7800 mg. One patient refused the challenge, but after a successful pistachio challenge, he ate one cashew unit at home without any reaction.

**TABLE 1:** Clinical and biological characteristics of the five patients.

	Patient 1	Patient 2	Patient 3	Patient 4	Patient 5
Age at first reaction with cashew nut, years	2.74	1.53	4.01	3.69	2.95
Age at the first consultation with allergist, years	2.9	1.94	4.07	3.85	8.01
Age at the final oral food challenge, years	5.2	6.1	NA	4.4	8.7
Sex	Male	Female	Male	Male	Female
Concomitant sensitization	Pistachio	Pistachio	Pistachio	Pistachio	Pistachio
Comorbidities	Mild asthma	No comorbidities	No comorbidities	Mild asthma, atopic dermatitis	Atopic dermatitis
Severity score (oFAAS‐5) of the reaction	3	4	4	5	4
Diameter of the wheal at diagnosis, mm	0	20	4	3	7
Cashew nut specific IgE at diagnosis, KU/L	0.84	2.49	0.66	0.47	0.36
r anaO3 at diagnosis	0.63	1.97	‐	‐	‐
Diameter of the wheal at follow‐up, mm	0	0	0	0	0
Cashew nut specific IgE at follow‐up, KU/L	0.12	0.13	0.1	0.1	0.1
r anaO3 at follow‐up, KU/L	0.10	0.10	0.10	0.10	0.10

Abbreviations: NA, non‐applicable: The patient three refused the oral food challenge; oFAAS, ordinal food allergy severity score.

**FIGURE 1:**
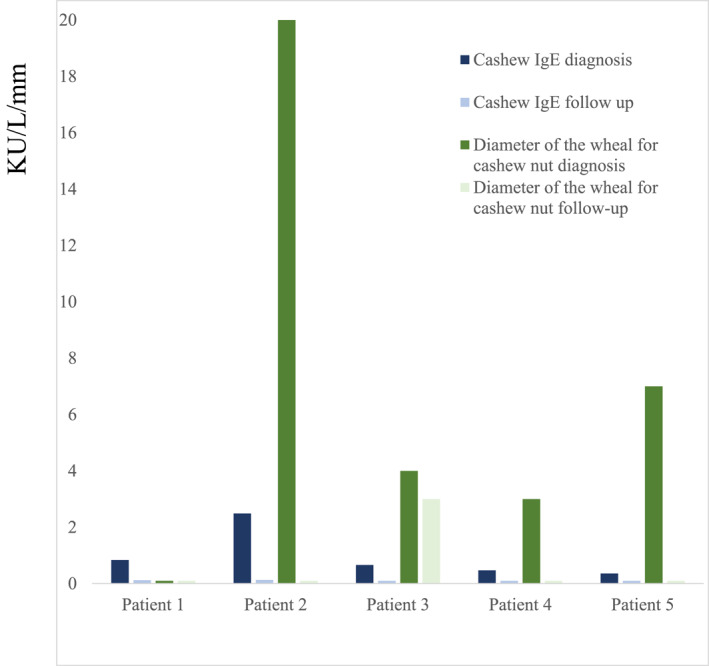
Cashew‐specific IgE and diameter of the wheal for cashew nut at diagnosis and during follow‐up in the five patients.

We reported the cases of five children who presented severe anaphylaxis to cashew nut and who spontaneously recovered after a mean follow‐up of 2.4 years [1–4]. As for peanut, ingestion of cashew is associated with a high rate of severe anaphylactic reactions,^4^ but in our cohort it seemed not to be correlated with persistence of the allergy. Oral immunotherapy (OIT) may help develop tolerance to cashew, as reported by Elizur et al.^5^ in a cohort of 50 children aged >4 years, who presented severe clinical reactions with cutaneous and biological sensitization. At the end of their protocol, 88% of the children were able to consume 4000 mg of cashew, and 94% ate more than 1200 mg. However, severe reactions were reported during OIT, with use of epinephrine during consultation in the emergency department. This tolerance was obtained by consuming cashew nut daily. Concerning our patients, cashew tolerance was achieved spontaneously after a cashew avoidance diet. In our cohort, data on recombinant Ana o 3 sensitization at diagnosis were not available for 3 patients, but all patients tested negative for recombinant Ana o 3 after follow‐up. We hypothesize that all patients were positive for recombinant Ana o 3 at diagnosis, as all had severe clinical anaphylaxis at the first reaction. Recombinant Ana o 3 is associated with severe anaphylaxis to cashew nut, with a specificity of 94%.^6^ In the cohort of Elizur et al., recombinant Ana o 3 was high at diagnosis in all patients and did not significantly decrease in control patients (Ana o 3‐ sIgE, median 8.3–5.9 kU/L, *p* = 0.5) but only in desensitized patients (median 3.7–1.6 kU/L, *p* < 0.001). In their study, the median follow‐up was 12 months (range, 3–57 months) in desensitized patients and 17 months (range, 5–44.1 months) in control patients, whereas in our cohort, the mean follow‐up was 28 months [12–48], which could require a longer follow‐up to monitor IgEs and SPTs. As described by Foong et al., tree nut‐specific IgE <2 KU/L may be predictive of resolution of allergy during follow‐up.^7^ However, in cashew allergy, there is a discordance between clinical and laboratory findings as specific IgEs are not always high in patients with severe anaphylaxis.^6^

To conclude, we propose that clinicians should continue to perform SPTs and monitor cashew‐specific IgEs even if the first reaction is severe, as it is possible that sensitization to cashew may spontaneously disappear. Oral food challenge can be then proposed if the laboratory parameters became negative, in order to confirm the recovery. Prospective studies will help confirm our hypothesis that CNA may resolve spontaneously.

## AUTHOR CONTRIBUTIONS

**Tamazoust Guiddir**: Conceptualization, investigation, methodology, validation, writing ‐ review & editing, writing ‐ original draft, formal analysis. **Audrey Siberil**: Methodology, writing ‐ original draft, investigation. **Francoise Lepape**: Methodology, investigation. **Marion Hacker**: Writing ‐ original draft, methodology, investigation. **Ariane Nemni**: Conceptualization, investigation, methodology, validation, writing ‐ review & editing, writing ‐ original draft, supervision.

## CONFLICT OF INTEREST STATEMENT

The authors declare that they have no conflicts of interest for this article.
